# Global View of Per Capita Daily Vitamin D Supply Estimates as Proxy Measures for Vitamin D Intake Data

**DOI:** 10.1002/jbm4.10547

**Published:** 2021-09-15

**Authors:** Kevin D. Cashman

**Affiliations:** ^1^ Cork Centre for Vitamin D and Nutrition Research, School of Food and Nutritional Sciences University College Cork Cork Ireland

**Keywords:** FAO FOOD BALANCE SHEET, FOOD SUPPLY, FORTIFICATION, GLOBAL, VITAMIN D

## Abstract

Nationally representative data on vitamin D intake can inform on the adequacy of dietary supply of vitamin D in a population, but such data is lacking for a majority of countries. Estimates of average per capita supply of vitamin D, as calculated using information from the Food and Agriculture Organisation of the United Nations (FAO) national food balance sheets (FBSs) can be used as proxy measures for vitamin D intake within a population. In the present work, FAO national FBSs (from 2004 to 2017) for 173 to 178 countries around the globe were used to generate such average per capita vitamin D supply estimates. For countries where food fortification with vitamin D was common, the estimates accounted for this. Using the 2004–2013 FBS data, there was a large range in average per capita vitamin D supply ranging from 0.3 (Ethiopia) to 17.8 (Maldives) μg/d. Globally, 40, 60, 70, four, two, and two countries had average per capita vitamin D supply estimates <1.5, 1.6–3, 3.1–5.5, 5.6–7.5, 7.6–10, and >10 μg/d, respectively. Pelagic fish was the major contributory food commodity (supplying 53%–86%) in countries with supply >7.6 μg/d. Median per capita vitamin D supply estimates for constituent countries within Africa, Americas, Asia, Europe, and Oceania were 1.4, 2.7, 2.8, 4.1, and 4.7 μg/d, respectively. These overall supply trends were mirrored in the newer, 2014–2017 FBS data. Fortification of milk and dairy or wheat flour with vitamin D had an important impact on the vitamin D supply estimates (average increments of 1.6 and 3.1 μg/d, respectively). Overall, the work showed how the per capita daily vitamin D supply estimates, as surrogate for vitamin D intake data, can highlight countries where inadequacy of supply may be of concern. It also shows how fortification of food with vitamin D can have an important impact on addressing low vitamin D intake. © 2021 The Author. *JBMR Plus* published by Wiley Periodicals LLC on behalf of American Society for Bone and Mineral Research.

## Introduction

In recent years there has been growing concern about vitamin D deficiency as a possible public health concern in some low‐income and lower‐middle–income countries (LMICs),^(^ ^1^ ^)^ which now sits alongside the more longstanding emphasis placed on the issue of vitamin D deficiency in higher‐income countries. In terms of prevalence and surveillance of vitamin D deficiency, where possible, ideally assessment of the vitamin D status of a population should be primarily based on recent nationally or sub‐nationally representative serum or plasma 25‐hydroxyvitamin D (25(OH)D) concentration data.^(^ ^2^ ^)^ Although estimates of the prevalence of vitamin D deficiency (based on serum 25(OH)D data) in representative population samples in the United States, Canada, and in some, but not all, parts of Europe are available,^(^ ^3^, ^4^, ^5^ ^)^ representative 25(OH)D data do not yet exist for many countries outside these higher‐income country settings. For example, a recent systematic review of vitamin D status in LMICs highlighted how nearly two‐thirds had no published studies with vitamin D data suitable for inclusion.^(^ ^6^ ^)^ The unavailability of representative 25(OH)D data for some middle‐income countries is also of note.^(^ ^7^, ^8^ ^)^

Nationally or regionally representative data on vitamin D intake in the population or in population subgroups may provide an additional source of information by which to determine the adequacy of vitamin D status in the population. An international working group, briefed with the assessment of the global prevalence and disease burden of vitamin D deficiency, recently emphasized that such vitamin D intake estimates could help to identify settings where a 25(OH)D assessment survey is warranted.^(^ ^2^ ^)^ Unfortunately, such intake data is also not available for many countries, irrespective of economic setting. In the absence of such intake data, we have recently shown that estimates of average per capita supply of vitamin D in LMICs, as calculated using information from the Food and Agriculture Organisation of the United Nations (FAO) national food balance sheets (FBSs), may be of use as proxy measures for vitamin D intake within the population.^(^ ^1^, ^6^ ^)^ These data can highlight a low supply of vitamin D owing to the limited availability of naturally rich food sources. It is also feasible to link such data with that from the Global Fortification Data Exchange online database^(^ ^9^ ^)^ to ascertain whether a country has vitamin D added to a staple food vehicle(s) on either a mandatory or voluntary basis.

Therefore, the objective of the present work was to use data from the FAO national FBSs for all available countries to provide for the first time a global mapping of average per capita daily vitamin D supply as proxy measures for vitamin D intake. In addition, for countries where mandatory food fortification with vitamin D was common, the vitamin D supply estimates accounted for this.

## Materials and Methods

### FAO FBSs from 2004 to 2013 and 2014 to 2017

The FAOSTAT database (Statistics Division, FAO, Rome, Italy; http://www.fao.org/faostat/en/#home) contains FBS data for approximately 173 to 178 countries around the globe (including special administrative regions [SARs] and some former countries), depending on the time frame. The FBSs from 1961 to 2013 are referred to as “old” FBSs^(^ ^10^ ^)^ and those from 2014 to 2017 are referred to as “new” FBSs,^(^ ^11^ ^)^ because their methodology in terms of use of a balancer variable in their calculations differed.^(^ ^12^ ^)^ For the present work, FAO FBSs for all available countries for the period 2004–2013 and 2014–2017, were downloaded as .csv files from the FAOSTAT database.^(^ ^10^, ^11^ ^)^ These FBSs provide overall per capita supply (as kg/year) for various food commodities, which were converted to g/d for ease of estimation of vitamin D supply in the present work.

### Estimation of average per capita vitamin D supply using FAO FBSs from 2004 to 2017

The general method of estimation of vitamin D supply using FAO FBSs has been outlined in detail elsewhere^(^ ^1^, ^6^, ^13^ ^)^; but in brief, the food commodities from the FBSs were assigned to their most appropriate match using data from the UK McCance and Widdowson's food composition database,^(^ ^14^ ^)^ and to a much more limited extent in the case of some fish species, the United States Department of Agriculture (USDA) food compositional database (FoodData Central [FDC]; https://fdc.nal.usda.gov/).^(^ ^15^ ^)^ This method assesses the majority of the selected commodities in their natural, nonprocessed state and assigns an average vitamin D compositional value to each food commodity grouping based on a range of vitamin D content values for individual foods within the database representative of that particular food subcategory; eg, demersal fish, pelagic fish, etc., under the fish grouping (see Supplemental Table S1). This allowed for the estimation of average per capita vitamin D supply (μg/d) for the periods 2004–2013 and 2014–2017, representing the most recent decade of data in the old FBSs as well as available new FBS data, respectively. Since the analyses for this work was completed, FAO FBSs for 2018 were released—these are not included, with the exception of those for India, which instigated fortification of milk (and vegetable oil) with vitamin D in 2018^(^ ^16^ ^)^ and thus this latest FBS data was used to examine the potential effect of this fortification on average per capita vitamin D supply.

The UK food compositional tables incorporate content data on vitamin D_3_ plus 25(OH)D_3_ multiplied by a bioactivity factor of five in its total vitamin D values for eggs, some meat, some fish, and animal fats.^(^ ^14^ ^)^ The USDA's FDC has recently reported 25(OH)D_3_ data for beef and a limited number of other foods,^(^ ^15^ ^)^ but does not include these data in total vitamin D values for the foods. These differences can impact the estimate that these animal foods make to the mean daily intake/supply of vitamin D.^(^ ^17^, ^18^ ^)^ Thus, average per capita vitamin D supply (μg/d) for the periods 2004–2013 and 2014–2017 were again estimated but by excluding the 25(OH)D_3_ data from the UK food compositional table data.

### Review and incorporation of data on national standards for addition of vitamin D to food

Although the FAO food balance sheet data will highlight low supply of vitamin D due to limited supply of naturally‐rich food sources, it will not capture the contribution that fortification of food with vitamin D would make to vitamin D supply in countries where this occurs. Therefore, the FAO balance sheet data was cross‐connected with data from the Global Fortification Data Exchange (https://fortificationdata.org/) in relation to the number of food vehicles with vitamin D fortification standards by country and the proportion of vehicle that is fortified, especially where fortification is voluntary.^(^ ^9^ ^)^ This identified mandatory or voluntary vitamin D fortification standards for maize flour, oil, rice, and/or wheat flour (Supplemental Table S2). Estimation of the likely contribution that vitamin D–fortified oil would make to average per capita vitamin D supply estimates, based on FAO FBS data, is problematic. For example, the FBSs contain data on numerous different oils but in many cases the food standards do not specify the type of oil other than vegetable oil or edible oil. In addition, some of the oils contained within the FBSs can be used for cooking. The retention of vitamin D in vegetable oil heated by different household cooking methods has been shown to be quite variable.^(^ ^19^, ^20^ ^)^ Therefore, with such uncertainties, for the present work vitamin D–fortified oils were not included in the estimation of the impact of vitamin D fortification of food on average per capita vitamin D supply.

Although the Global Fortification Data Exchange does not capture data on milk within its listing of food vehicles,^(^ ^9^ ^)^ it is possibly the most commonly fortified food for vitamin D in many high‐income countries.^(^ ^2^ ^)^ Thus, for the present work, information on high‐income countries with a policy of vitamin D fortification of fluid milk products was taken from a recent review.^(^ ^21^ ^)^

In all cases, irrespective of the food vehicle(s) used, emphasis was placed on mandatory fortification over voluntary fortification. The advantages of mandatory versus voluntary fortification of food with vitamin D, in terms of increasing the dietary intake of the vitamin in the general population in low‐middle–income and high‐income countries, have been reviewed recently.^(^ ^2^ ^)^ In addition, the level of uptake of voluntary fortification is unclear.^(^ ^9^ ^)^ Even in the case of mandatory fortification, although data on the percentage of industrially processed foods that are fortified with selected micronutrients are available, data in relation to vitamin D is sparse.^(^ ^9^ ^)^ Thus, this work made the assumption that all data within the FBSs pertaining to wheat and wheat products, as well as maize and maize products, were as flour, and therefore available for fortification with vitamin D. Data from the Global Fortification Data Exchange on the percentage of flour that is fortified with vitamin D in each of the countries was used in the calculations.

In the case of milk fortification with vitamin D, although the United States and Finland have voluntary fortification, it is in essence universal and thus closer to the practice of mandatory fortification, as seen in Canada.^(^ ^21^ ^)^ Because the FAO FBSs provide data on average per capita supply of milk (excluding butter) in its most nonprocessed form, this data was cross‐connected with data on per capita consumption of milk (and other key milk products covered under fortification policy; eg, yoghurt and/or sourmilk, as per national standards) in the United States, Canada, Finland, and Sweden.^(^ ^22^ ^)^ The percentage of overall milk supply consumed as these products was estimated.

The year that the fortification of the various food vehicles with vitamin D was instigated and the levels used (both as per Supplemental Table S2), as well as the % of overall supply in the case of milk, was applied to the relevant FAO FBSs and the average per capita vitamin D supply (μg/d) estimated.

### Comparison of per capita vitamin D supply estimates using FAO FBSs with vitamin D intake estimates from national nutrition surveys

Vitamin D intake data from national (or sub‐national) nutrition surveys from 2004 onward from around the globe were sourced using a PubMed search and searches for key survey reports. Ten such surveys were identified.^(^ ^23^, ^24^, ^25^, ^26^, ^27^, ^28^, ^29^, ^30^, ^31^, ^32^, ^33^ ^)^ Mean vitamin D intakes from food sources only, excluding contribution of vitamin D supplements, were prioritized because the FAO FBSs approach does not capture supplemental vitamin D. The per capita vitamin D supply estimates from the same year(s) of the national surveys (based on inclusion or exclusion of the bioactivity‐adjusted 25(OH)D food content as pertained in the individual nutrition surveys) were used as the comparator in a Bland‐Altman analysis.^(^ ^34^ ^)^

## Results

### Comparison of per capita vitamin D supply estimates using FAO FBSs with vitamin D intake estimates from national nutrition surveys

**Fig 1:**
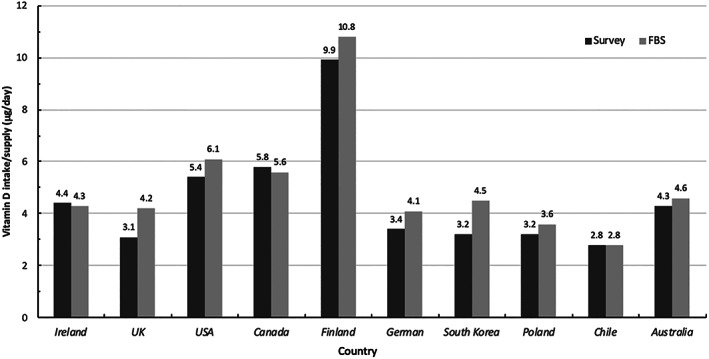
Comparison of per capita vitamin D supply estimates using FAO FBSs with vitamin D intake estimates from national nutrition surveys. FBS = food balance sheet.

The comparison of estimates of average daily vitamin D supply and vitamin D intakes from 10 countries,^(^ ^23^, ^24^, ^25^, ^26^, ^27^, ^28^, ^29^, ^30^, ^31^, ^32^, ^33^ ^)^ using the FAO FBS approach and data from national nutrition surveys, respectively, is shown in Fig. 1. Bland‐Altman analysis showed that overall there was moderately good agreement between the vitamin D estimates (*p* = 0.592), with those from the FAO FBS approach having a mean ± SD positive bias of 0.5 ± 0.5 μg/d (data not shown). The bias at a country‐level in the FBS‐derived estimates relative to those from national/sub‐national nutrition surveys ranged from −0.2 to 1.3 μg/d.

### Average per capita vitamin D supply estimates from natural food sources for 2004–2013 and 2014–2017

The average per capita vitamin D supply estimates for the periods 2004–2013 and 2014–2017 are shown in Table 1. It should be noted again that these estimates are from vitamin D naturally present in the foods and do not account for vitamin D added through fortification. Data from the “old” FBSs (2004–2013), and not including 25(OH)D compositional data, shows that there was a large range in average per capita vitamin D supply estimates from 0.3 μg/d (Ethiopia) to 17.8 μg/d (Maldives). Forty countries had average per capita vitamin D supply estimates <1.5 μg/d. A further 60 and 70 countries had average per capita vitamin D supply estimates in the ranges 1.6–3 and 3.1–5.5 μg/d, respectively. Only four, two, and two countries had average per capita vitamin D supply estimates in the range 5.6–7.5, 7.6–10, and >10 μg/d, respectively. In each of the four countries with intakes ≥7.6 μg/d, pelagic fish was the food commodity making the major contribution (53%–86%, depending on the country) to the vitamin D supply estimates, with fish liver oil also being an important contributor in the case of Iceland (data not shown).

**Table 1:** Average Per Capita Vitamin D Supply Estimates From Natural Food Sources for All Countries With Available Food Balance Sheet Data (2004–13 and 2014–17)

Country/region	Vitamin D supply (μg/d)							
	2004–2013 FBS data				2014–2017 FBS data			
	Total vitamin D estimate^a^		Less 25(OH)D		Total vitamin D estimate^a^		Less 25(OH)D	
	Mean	SD	Mean	SD	Mean	SD	Mean	SD
Afghanistan	0.5	0.0	0.4	0.0	0.5	0.0	0.4	0.0
Albania	2.9	0.3	2.6	0.2	3.5	0.1	3.1	0.1
Algeria	1.8	0.1	1.6	0.1	1.9	0.0	1.6	0.0
Angola	1.9	0.3	1.7	0.3	3.0	0.6	2.8	0.5
Antigua and Barbuda	4.1	0.4	3.8	0.3	4.0	0.1	3.7	0.1
Argentina	3.2	0.3	2.8	0.2	3.7	0.0	3.2	0.0
Armenia	2.4	0.5	2.1	0.4	3.2	0.0	2.8	0.0
Australia	4.3	0.3	3.9	0.2	4.8	0.0	4.3	0.0
Austria	5.0	0.2	4.5	0.1	4.9	0.1	4.3	0.0
Azerbaijan	1.7	0.3	1.5	0.2	2.0	0.1	1.7	0.1
Bahamas	4.7	0.2	4.3	0.2	4.2	0.1	3.8	0.1
Bangladesh	2.5	0.3	2.3	0.2	3.4	0.1	3.1	0.1
Barbados	5.7	0.5	5.2	0.4	6.2	0.1	5.7	0.1
Belarus	5.2	0.4	4.6	0.4	5.0	0.1	4.4	0.1
Belgium	5.9	1.5	5.4	1.4	5.0	0.1	4.4	0.1
Belize	2.7	0.3	2.4	0.3	2.1	0.1	1.9	0.0
Benin	1.1	0.1	1.0	0.1	1.9	0.2	1.7	0.1
Bermuda	4.5	0.6	4.0	0.6	‐	‐	‐	‐
Bolivia (Plurinational State of)	1.7	0.1	1.5	0.1	1.9	0.1	1.7	0.0
Bosnia and Herzegovina	1.9	0.1	1.8	0.1	2.1	0.0	1.9	0.0
Botswana	1.3	0.1	1.2	0.1	1.5	0.1	1.4	0.1
Brazil	3.0	0.4	2.6	0.3	3.3	0.0	2.9	0.0
Brunei Darussalam	3.8	0.3	3.4	0.3	‐	‐	‐	‐
Bulgaria	3.0	0.1	2.6	0.1	3.0	0.0	2.7	0.0
Burkina Faso	1.1	0.1	0.9	0.1	1.3	0.0	1.2	0.0
Cabo Verde	2.7	0.4	2.5	0.4	2.1	0.0	1.9	0.0
Cambodia	4.8	0.8	4.3	0.7	5.5	0.1	5.0	0.0
Cameroon	2.3	0.2	2.1	0.2	2.3	0.3	2.1	0.3
Canada	4.5	0.1	4.0	0.1	4.8	0.0	4.3	0.0
Central African Republic	1.8	0.1	1.6	0.1	1.8	0.0	1.6	0.0
Chad	1.0	0.1	0.9	0.1	1.7	0.0	1.6	0.0
Chile	3.3	0.2	3.0	0.2	3.4	0.1	2.9	0.1
China	4.9	0.4	4.3	0.4	6.0	0.0	5.2	0.0
China, Hong Kong SAR	5.6	0.6	5.0	0.6	7.4	0.2	6.5	0.2
China, Macao SAR	5.1	0.5	4.5	0.4	6.0	0.2	5.2	0.2
China, mainland	4.9	0.4	4.3	0.4	6.0	0.0	5.2	0.0
China, Taiwan Province of	4.3	0.2	3.8	0.2	4.1	0.1	3.6	0.1
Cote d'Ivoire	1.8	0.3	1.7	0.3	2.7	0.0	2.5	0.0
Colombia	2.7	0.2	2.3	0.2	3.2	0.0	2.8	0.0
Congo	2.9	0.4	2.7	0.4	3.3	0.1	3.0	0.1
Costa Rica	3.2	0.4	2.8	0.4	4.4	0.3	3.9	0.3
Croatia	4.5	0.3	4.0	0.3	4.5	0.2	4.0	0.2
Cuba	2.3	0.3	2.0	0.2	2.5	0.0	2.2	0.0
Cyprus	3.9	0.2	3.5	0.2	3.7	0.0	3.3	0.0
Czechia	4.3	0.2	3.8	0.2	3.7	0.0	3.3	0.0
DPR of Korea	0.7	0.1	0.6	0.0	0.7	0.0	0.6	0.0
Denmark	6.1	0.4	5.4	0.4	5.5	0.1	4.9	0.1
Djibouti	0.9	0.1	0.8	0.1	1.9	2.0	1.8	1.8
Dominica	4.7	0.6	4.3	0.6	5.4	0.2	5.0	0.2
Dominican Republic	2.2	0.2	2.0	0.2	2.7	0.1	2.4	0.1
Ecuador	2.4	0.3	2.2	0.2	2.7	0.2	2.4	0.2
Egypt	3.0	0.6	2.7	0.5	3.9	0.1	3.5	0.1
El Salvador	2.2	0.2	1.9	0.2	2.5	0.1	2.2	0.1
Estonia	3.8	0.3	3.4	0.2	3.9	0.1	3.5	0.1
Eswatini	1.1	0.7	1.0	0.6	0.9	0.1	0.8	0.1
Ethiopia	0.3	0.0	0.3	0.0	0.3	0.0	0.3	0.0
Fiji	5.7	0.4	5.2	0.3	4.4	0.0	4.1	0.0
Finland	7.8	0.3	7.1	0.3	7.9	0.2	7.2	0.2
France	5.7	0.2	5.1	0.1	5.4	0.1	4.9	0.1
French Polynesia	7.1	0.1	6.5	0.1	7.0	0.2	6.3	0.2
Gabon	4.8	0.3	4.5	0.3	4.8	0.3	4.4	0.2
Gambia	3.5	0.4	3.2	0.4	3.4	0.5	3.2	0.5
Georgia	2.5	0.3	2.2	0.2	2.4	0.1	2.1	0.1
Germany	4.6	0.1	4.1	0.1	4.5	0.1	4.0	0.1
Ghana	4.6	0.4	4.2	0.4	4.2	0.2	3.8	0.2
Greece	3.9	0.2	3.5	0.2	3.7	0.1	3.3	0.1
Grenada	5.5	0.3	5.0	0.3	4.8	0.2	4.3	0.2
Guatemala	2.1	0.1	1.7	0.1	2.0	0.0	1.7	0.0
Guinea	1.2	0.3	1.1	0.2	1.9	0.1	1.7	0.0
Guinea‐Bissau	0.5	0.0	0.4	0.0	0.8	0.6	0.7	0.5
Guyana	1.6	0.2	1.5	0.1	1.5	0.5	1.4	0.5
Haiti	0.8	0.1	0.7	0.1	0.9	0.1	0.8	0.1
Honduras	1.7	0.2	1.5	0.2	1.3	0.0	1.2	0.0
Hungary	3.9	0.3	3.4	0.2	3.7	0.2	3.3	0.1
Iceland	14.2	0.7	13.3	0.6	14.7	0.0	13.7	0.0
India	1.1	0.1	1.0	0.1	1.4	0.1	1.3	0.1
Indonesia	3.6	0.5	3.3	0.4	5.2	0.5	4.7	0.4
Iran (Islamic Republic of)	2.3	0.2	2.1	0.1	2.9	0.1	2.6	0.1
Iraq	1.0	0.4	0.9	0.3	1.3	0.2	1.1	0.2
Ireland	4.6	0.2	4.1	0.2	5.3	0.3	4.8	0.3
Israel	4.5	0.5	4.1	0.5	5.6	0.2	5.1	0.2
Italy	4.7	0.1	4.1	0.0	4.6	0.1	4.1	0.1
Jamaica	3.1	0.2	2.9	0.2	2.8	0.1	2.6	0.1
Japan	6.2	0.2	5.5	0.2	6.0	0.0	5.3	0.0
Jordan	1.7	0.2	1.6	0.1	1.6	0.1	1.5	0.0
Kazakhstan	3.0	0.3	2.7	0.3	3.0	0.0	2.7	0.0
Kenya	1.2	0.1	1.1	0.1	1.1	0.0	1.0	0.0
Kiribati	8.3	2.0	7.6	1.8	8.8	4.3	8.1	3.9
Kuwait	4.0	0.3	3.6	0.3	3.4	0.1	3.0	0.1
Kyrgyzstan	1.7	0.1	1.6	0.1	2.0	0.9	1.8	0.7
Lao PDR	3.1	0.3	2.8	0.3	4.4	0.1	4.0	0.1
Latvia	5.7	0.9	5.1	0.8	5.4	0.1	4.8	0.1
Lebanon	2.1	2.2	1.9	2.0	1.6	0.1	1.4	0.1
Lesotho	0.5	0.0	0.4	0.0	0.7	0.1	0.7	0.1
Liberia	0.7	0.1	0.7	0.1	1.1	0.1	1.0	0.1
Lithuania	9.1	0.8	8.3	0.7	8.7	0.2	7.9	0.2
Luxembourg	5.0	0.5	4.5	0.4	5.3	0.1	4.7	0.1
Madagascar	0.9	0.1	0.8	0.1	0.7	0.0	0.6	0.0
Malawi	1.1	0.2	1.0	0.2	1.7	0.2	1.6	0.2
Malaysia	6.5	0.5	5.9	0.5	6.7	0.2	6.0	0.2
Maldives	19.4	6.7	17.8	6.2	21.9	3.9	20.1	3.6
Mali	1.8	0.2	1.7	0.2	1.5	0.1	1.4	0.1
Malta	6.1	0.3	5.4	0.2	5.8	0.1	5.2	0.1
Mauritania	2.1	0.1	2.0	0.1	2.0	0.1	1.9	0.1
Mauritius	3.2	0.3	2.9	0.3	3.5	0.0	3.1	0.0
Mexico	3.7	0.2	3.2	0.1	4.6	0.1	4.0	0.1
Mongolia	1.5	0.1	1.4	0.1	1.9	0.1	1.8	0.1
Montenegro	3.7	0.5	3.4	0.4	4.5	0.2	4.0	0.2
Morocco	2.8	0.6	2.5	0.6	4.0	0.3	3.6	0.3
Mozambique	0.5	0.2	0.5	0.1	1.0	0.0	0.9	0.0
Myanmar	4.1	1.1	3.7	1.0	6.4	0.1	5.8	0.1
Namibia	2.0	0.3	1.8	0.3	2.0	0.0	1.9	0.0
Nepal	0.7	0.0	0.6	0.0	0.9	0.0	0.8	0.0
Netherlands	5.8	0.4	5.2	0.4	5.0	0.0	4.5	0.0
Netherlands Antilles (former)	4.0	0.2	3.7	0.2	‐	‐	‐	‐
New Caledonia	4.8	0.2	4.4	0.2	4.4	0.1	3.9	0.1
New Zealand	5.2	0.4	4.7	0.4	4.3	0.1	3.9	0.1
Nicaragua	1.5	0.2	1.3	0.1	1.9	0.1	1.7	0.1
Niger	0.9	0.1	0.8	0.1	0.7	0.1	0.6	0.0
Nigeria	2.0	0.3	1.8	0.3	1.6	0.2	1.5	0.2
North Macedonia	2.4	0.1	2.2	0.1	2.2	0.1	2.0	0.0
Norway	5.1	0.3	4.6	0.3	5.5	0.2	5.0	0.2
Oman	3.6	0.5	3.3	0.4	3.7	0.1	3.4	0.1
Pakistan	1.3	0.1	1.2	0.1	1.3	0.0	1.2	0.0
Panama	2.9	0.2	2.6	0.2	3.2	0.0	2.9	0.0
Paraguay	3.3	0.1	2.8	0.0	3.0	0.4	2.6	0.3
Peru	3.6	0.3	3.3	0.3	4.2	0.2	3.8	0.2
Philippines	5.4	0.3	4.9	0.3	4.8	0.1	4.3	0.1
Poland	4.1	0.2	3.7	0.2	3.9	0.0	3.5	0.0
Portugal	5.5	0.4	4.9	0.4	5.3	0.1	4.9	0.1
Republic of Korea	5.3	0.2	4.7	0.1	5.2	0.1	4.7	0.1
Republic of Moldova	3.4	0.2	3.0	0.2	3.0	0.1	2.7	0.1
Romania	3.6	0.2	3.2	0.1	3.8	0.0	3.4	0.0
Russian Federation	4.9	0.4	4.4	0.4	4.9	0.2	4.3	0.1
Rwanda	0.3	0.1	0.3	0.1	0.6	0.0	0.6	0.0
Saint Kitts and Nevis	3.2	0.2	3.0	0.2	2.7	0.0	2.5	0.0
Saint Lucia	4.6	0.5	4.2	0.5	4.8	0.1	4.4	0.1
Saint Vincent and the Grenadines	2.9	0.1	2.6	0.1	2.7	0.0	2.5	0.0
Samoa	8.2	0.5	7.5	0.5	8.2	0.2	7.5	0.2
Sao Tome and Principe	3.6	0.1	3.3	0.1	3.8	0.2	3.5	0.2
Saudi Arabia	2.4	0.4	2.2	0.4	2.8	0.0	2.6	0.0
Senegal	3.9	0.4	3.6	0.4	2.7	0.1	2.5	0.1
Serbia	2.7	0.3	2.4	0.2	3.1	0.1	2.7	0.1
Serbia and Montenegro	3.1	0.0	2.7	0.0	‐	‐	‐	‐
Sierra Leone	4.1	0.6	3.8	0.6	3.6	0.1	3.3	0.1
Slovakia	3.5	0.2	3.0	0.1	3.4	0.2	3.0	0.2
Slovenia	3.9	0.2	3.5	0.2	3.7	0.1	3.3	0.1
Solomon Islands	2.8	0.3	2.5	0.2	4.2	0.4	3.8	0.4
South Africa	2.3	0.1	2.0	0.1	2.2	0.1	2.0	0.1
Spain	5.8	0.2	5.2	0.2	5.8	0.2	5.1	0.2
Sri Lanka	3.9	0.4	3.5	0.4	4.9	0.0	4.5	0.0
Sudan	1.0	0.0	1.0	0.0	0.9	0.0	0.8	0.0
Sudan (former)	1.2	0.0	1.1	0.0	‐	‐	‐	‐
Suriname	1.9	0.4	1.7	0.4	3.9	0.7	3.6	0.6
Sweden	6.0	0.2	5.4	0.2	5.9	0.1	5.3	0.1
Switzerland	4.5	0.1	4.0	0.1	4.5	0.1	4.0	0.0
Tajikistan	0.5	0.1	0.5	0.1	0.6	0.0	0.5	0.0
Thailand	4.6	0.2	4.1	0.1	4.4	0.4	3.9	0.4
Timor‐Leste	0.7	0.2	0.6	0.2	1.0	0.1	0.9	0.1
Togo	1.1	0.2	1.0	0.2	1.6	0.1	1.4	0.1
Trinidad and Tobago	2.9	0.2	2.7	0.2	3.0	0.1	2.7	0.1
Tunisia	2.9	0.1	2.6	0.1	2.8	0.1	2.5	0.1
Turkey	2.5	0.1	2.3	0.1	2.3	0.0	2.1	0.0
Turkmenistan	2.3	0.1	2.1	0.1	2.7	0.1	2.1	0.0
Uganda	2.2	0.2	2.0	0.2	2.2	0.2	2.0	0.2
Ukraine	4.7	0.4	4.1	0.4	3.8	0.3	3.3	0.2
United Arab Emirates	3.1	0.4	2.9	0.4	3.9	0.2	3.5	0.1
United Kingdom	4.2	0.1	3.7	0.1	3.9	0.0	3.5	0.0
United Republic of Tanzania	1.2	0.1	1.1	0.1	1.2	0.1	1.1	0.1
United States of America	5.0	0.1	4.4	0.0	5.1	0.0	4.5	0.0
Uruguay	3.1	0.4	2.7	0.4	3.3	0.2	2.9	0.2
Uzbekistan	1.3	0.1	1.1	0.1	2.0	0.1	1.8	0.1
Vanuatu	4.6	0.5	4.2	0.5	3.3	0.1	3.0	0.1
Venezuela (Bolivarian Republic of)	3.0	0.2	2.7	0.2	2.6	0.2	2.4	0.2
Viet Nam	3.2	0.5	2.8	0.5	4.1	0.3	3.6	0.2
Yemen	1.0	0.4	0.9	0.4	1.0	0.2	0.9	0.2
Zambia	1.4	0.1	1.2	0.1	2.3	0.2	2.0	0.2
Zimbabwe	0.7	0.1	0.6	0.1	1.0	0.1	0.9	0.1

25(OH)D = 25‐hydroxyvitamin D; DPR = Democratic People's Republic; FBS = food balance sheet; PDR = People's Democratic Republic; SAR = Special Administrative Region.

^a^ Includes bioactivity‐adjusted 25(OH)D content.

Data from the “new” FBSs (2014–2017), and not including 25(OH)D compositional data, shows similar trends to that from the earlier time frame; ie, a large range in average per capita vitamin D supply estimates from 0.3 μg/d (Ethiopia) to 20.1 μg/d (Maldives); 32, 57, 73, 7, two, and two countries had average per capita vitamin D supply estimates in the range < 1.5, 1.6–3, 3.1–5.5, 5.6–7.5, 7.6–10, and >10 μg/d, respectively.

Using the data from both sets of FBSs and arranging the countries by world geographic regions showed that median (25th and 75th percentile) per capita vitamin D supply estimates for those countries within Africa, the Americas, Asia, Europe, and Oceania was 1.4 (0.9, 4.5), 2.7 (2.0, 5.2), 2.8 (1.5, 17.8), 4.1 (3.4, 13.3), and 4.7 (4.2, 7.6) μg/d, respectively, based on the 2004–2013 data, and 1.7 (1.0, 4.4), 2.8 (2.3, 5.7), 3.0 (1.7, 20.1), 4.0 (3.3, 13.7), and 4.1 (3.9, 8.1) μg/d, respectively, based on the 2014–2017 data.

For 127 countries/SARs (73%), the differences in average per capita vitamin D supply estimates between 2004 and 2013 and 2014 and 2017 were minor to modest (increase or decrease of <0.5 μg/d), with 14 countries having an increase or decrease of ≥1 μg/d. The Maldives and Myanmar had increases of ~2 μg/d, which are related to changes in per capita pelagic or freshwater fish supply (as major contributors to vitamin D supply) over the period (see Supplemental Fig. S1).

### Impact of inclusion of bioactivity‐adjusted 25(OH)D compositional data in the average per capita vitamin D supply estimates

Data from the 2004–2013 FBSs shows that the increment in average per capita vitamin D supply estimates when food 25(OH)D content and a bioactivity factor of 5 was included in the compositional data was in the range 0–0.1, 0.1–0.19, 0.2–0.29, 0.3–0.39, and 0.4–0.49 μg/d in 22, 30, 31, 32, and 22 countries, respectively. The increment in average per capita vitamin D supply estimates upon inclusion of bioactivity‐adjusted food 25(OH)D content was between 0.5 and 0.9 μg/d in 41 countries/SARs, and one country (Maldives) had an increment of 1.6 μg/d, much of which could be accounted for by pelagic fish consumption. The number of countries with an increment of ≥0.5 μg/d was 40 when the 2014–2017 FBS data was used (with the Maldives again with the greatest increment, 1.8 μg/d) (data not shown).

### Impact of fortification on the average per capita vitamin D supply estimates

The impact of vitamin D fortification of key food vehicles on average per capita vitamin D supply estimates (over the period of “new” FBSs; ie, 2014–2017) in those countries where it is common, both excluding and including the bioactivity‐adjusted food 25(OH)D content, is shown in Table 2. The average per capita vitamin D supply estimates for the same countries just using natural foods (excluding and including the bioactivity‐adjusted food 25(OH)D content) is also presented for comparison purposes.

**Table 2:** Per Capita Vitamin D Supply Estimates (Including and Excluding Bioactivity‐Adjusted 25(OH)D Content) for Countries Where Fortification of Food Is Common

Country	Estimated vitamin D supply (μg/d) [2014–2017]							
	Natural foods only				Including fortified foods			
	Total vitamin D estimate^a^		Less 25(OH)D		Total vitamin D estimate^a^		Less 25(OH)D	
	Mean	SD	Mean	SD	Mean	SD	Mean	SD
Finland	7.9	0.2	7.2	0.2	10.8	0.2	10.4	0.2
USA	5.1	0.0	4.5	0.0	7.2	1.1	6.7	1.1
Canada	4.8	0.1	4.3	0.0	6.2	0.1	5.9	0.1
Sweden	6.0	0.1	5.3	0.1	6.3	0.3	5.8	0.3
Jordan	1.6	0.1	1.5	0.1	5.9	0.2	5.8	0.2
Kuwait	3.4	0.1	3.0	0.1	6.2	1.9	6.0	1.9
United Arab Emirates	3.9	0.2	3.5	0.1	6.7	1.9	6.5	1.9
Saudi Arabia	2.8	0.0	2.6	0.0	5.5	1.8	5.4	1.8

25(OH)D = 25‐hydroxyvitamin D.

^a^ Based on the UK's McCance & Widdowson's Food Compositional Tables,^(^ ^14^ ^)^ includes bioactivity‐adjusted 25(OH)D content.

The increment in vitamin D supply estimates due to food fortification, and accounting for bioactivity‐adjusted food 25(OH)D content of natural foods, was higher for the four countries in the Middle East region (range, 2.7–4.2 μg/d) than for the four countries in the European/North American region (range, 0.3–2.8 μg/d). The fortification vehicle in the former was wheat flour, whereas it was largely milk and dairy for the latter. Even though the average increment in the four European/North American countries (1.6 μg/d) was almost half that of the four countries in the Middle East region (3.1 μg/d), the average overall supply was higher in the former (7.6 versus 6.1 μg/d, respectively) due to having double the vitamin D supply from natural food sources versus that in the Middle East region countries.

The average per capita vitamin D supply estimate for India based on the 2018 FBS data, and including vitamin D fortification of milk at a level of 6.25 μg/L^(^ ^16^ ^)^ together with an assumed 100% uptake of fortification by producers, was 3.1 μg/d. The equivalent vitamin D supply estimate was 1.5 μg/d, if fortification was not included.

### Global pattern of average per capita vitamin D supply estimates

**Fig 2:**
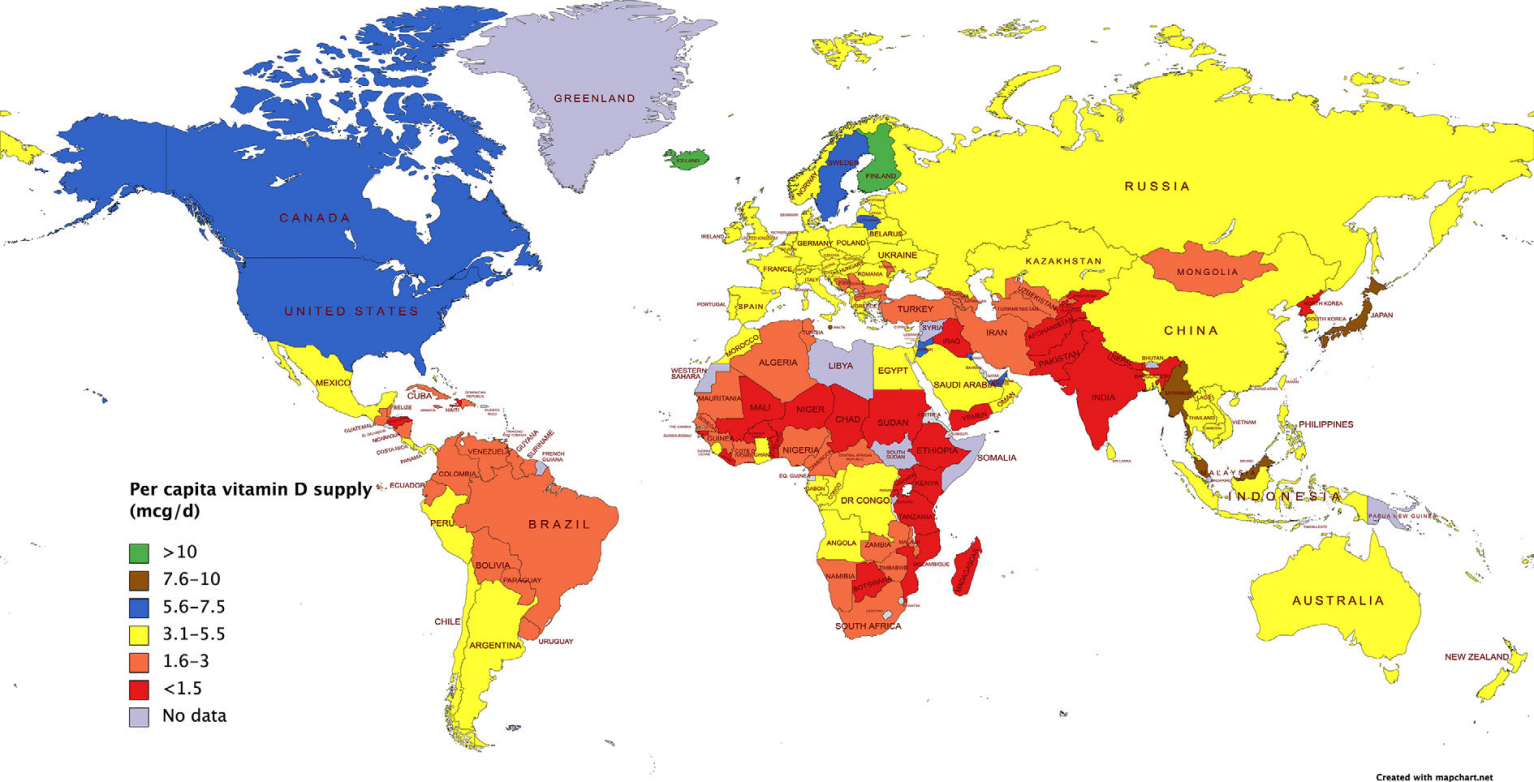
Global map of per capita vitamin D supply estimated using FAO FBS data. FBS = food balance sheet.

Based on the new food balance sheets data (2014–2017) and accounting for the contribution of food fortification (but not including the bioactivity‐adjusted food 25(OH)D content of natural foods because this is at present only favored by a minority of countries in the intake estimates), the individual countries falling into bands of average per capita vitamin D supply are shown in Fig. 2.

In general, although much of South America and Meso‐America had vitamin D supply estimates in the range 1.6 to 5.5 μg/d, the United States and Canada had vitamin D supply estimates in the range 5.6 to 7.5 μg/d. Much of Europe and Oceania had vitamin D supply estimates in the range 3.1 to 5.5 μg/d. The vitamin D supply estimates in Africa were variable but a majority in the range <1.5 to 3 μg/d, and a minority were in the range 3.1 to 5.5 μg/d (and a few African countries had no data available). Likewise, the vitamin D supply estimates in Asia were variable in the range < 1.5 to 10 μg/d, with a majority in the range <1.5 to 5.5 μg/d. Eastern Asia and South‐East Asia in general had vitamin D supply estimates in the range 3.1 to 5.5 μg/d, whereas Central Asia had <1.5 to 5.5 μg/d. Southern and Western Asia had much more variable vitamin D supply estimates with as low as <1.5 μg/d to as high as 10 μg/d.

The per capita daily supply of all fish combined, all meats combined, eggs, and dairy (as key natural food contributors to vitamin D supply) in the Americas, Europe, Africa, Asia, and Oceania is shown in Supplemental Fig. S2.

## Discussion

This work provides for the first time a global mapping of average per capita daily vitamin D supply estimates. It is important to re‐emphasize that these supply estimates, based on the FAO FBS approach and not derived via quantitative food and nutrient intake data from nationally representative surveys, are intended as a surrogate for vitamin D intake data. Unfortunately data on vitamin D intakes for a majority of countries globally, especially representative data, are very limited, if not largely absent,^(^ ^2^ ^)^ and although cognizant of the limitations of the present approach, the supply estimates provide some insight into the potential scale of inadequacy of vitamin D intake globally. For some countries this inadequacy of vitamin D intake will be of negligible impact on population vitamin D status and prevalence of vitamin D deficiency, owing to an abundance of UVB radiation available for the production of vitamin D in the skin. Yet for other countries/regions, the inadequacy of vitamin D intake, when coupled with low availability of UVB and/or avoidance of UVB exposure by a population subgroup(s), will have a material impact on vitamin D status and prevalence of vitamin D deficiency.

In the present work there was a large range in vitamin D supply estimates globally, with Ethiopia having the lowest (0.3 μg/d) and the Maldives having the highest (~20 μg/d). Unfortunately, representative data on vitamin D intakes in these two countries are not available, but data from smaller available studies support these estimates. For example, a cross‐sectional study of women in southern Ethiopia (mean age, 31 years; *n* = 202), although not providing a quantitative estimate of vitamin D intake, reported that the participants had almost no dietary sources of vitamin D.^(^ ^35^ ^)^ A cross‐sectional study of preschool children (*n* = 248) from southern Sri Lanka, a neighbor to the Maldives within the Indian Ocean, reported relatively high average daily vitamin D intakes for boys and girls aged 0 to 60 months (average of reported medians, 18 μg/d).^(^ ^36^ ^)^ Within the entire global collection of countries included in the present work, there were 10 with relatively recent nationally representative vitamin D intake data available. The present FAO FBS‐derived vitamin D supply estimates for these 10 countries were found to be in moderately good agreement with their reported vitamin D intakes, supporting their use as surrogates.

The vitamin D supply estimates are likely to be most informative for those countries where UVB‐induced cutaneous synthesis is limited or absent, irrespective of underlying reason. In general, for countries at above 37 degrees of latitude there will be a “vitamin D winter” period during which UVB availability will be limited or absent for a certain number of months.^(^ ^37^ ^)^ Within the Northern Hemisphere, although much of the United States and Canada fall into this latitudinal zone, the widespread practice of vitamin D fortification of food in these two countries helps to ensure moderately good vitamin D supply estimates (5.6–7.5 μg/d), not accounting for the contribution of vitamin D–containing supplement use within the population. Consequently, both countries have a relatively low prevalence of vitamin D deficiency as defined by a serum 25(OH)D threshold of <30 nmol/L^(^ ^38^ ^)^) at 5% and 7.4%, respectively, but 23.3% and 36.8%, respectively,^(^ ^3^, ^4^ ^)^ using the alternate suggested serum 25(OH)D threshold of 50 nmol/L.^(^ ^39^ ^)^ Vitamin D intakes of 4.7 μg/d and 7.5 μg/d have been shown to maintain serum 25(OH)D ≥25 nmol/L (as the minimal suggested threshold internationally) in 90% and 95% of individuals, respectively.^(^ ^40^ ^)^ The US Institute of Medicine established an estimated average requirement (EAR) intake value for vitamin D of 10 μg/d, which ensures that 50% of individuals would maintain serum 25(OH)D ≥40 nmol/L during wintertime.^(^ ^38^ ^)^ The average per capita supply estimates for the United States and Canada in the present work would predict a majority of individuals in these two countries will fail to meet this EAR of 10 μg/d from food sources (including fortified foods) alone. Although the latitudinal footprint of Europe is broadly similar to that of the United States and Canada, most European countries do not have widespread vitamin D fortification of food and their vitamin D supply estimates from natural food sources are in the range of 3.1 to 5.5 μg/d. The prevalence of vitamin D deficiency for Europe is 12% on average.^(^ ^5^ ^)^ In the Southern hemisphere, Chile and Argentina have significant portions of the country residing above 37 degrees of latitude. Both have vitamin D supply estimates from natural food sources in the range of 3.1 to 5.5 μg/d, and reported prevalence of vitamin D deficiency of 14% to 26% overall and higher in the Southern regions of the country (higher latitudes).^(^ ^41^, ^42^ ^)^ These Northern and Southern hemisphere countries residing at >37 degrees of latitude broadly track to upper‐middle–income to high‐income settings and as such have relatively good supply of foods of animal origin, key providers of vitamin D in the food chain.

The monthly changes in UV Index (UVI) over the course of a year in selected countries around the world at a variety of latitudes have been provided by the World Health Organization (WHO),^(^ ^43^ ^)^ and we have previously used this data as a crude proxy for UVB availability estimates in countries at similar latitudes.^(^ ^6^ ^)^ In general, the higher the UVI, the greater the availability of UVB radiation. Both the Northern and Southern hemisphere‐based countries in the latitudinal zone between 37 degrees north and 37 degrees south have much higher UVI scores throughout the year (8 to 12; ie, very high to extreme) compared to their equivalents at >37 degrees of latitude (2 to 6; ie, low to moderate). In terms of per capita vitamin D supply from food, the estimates were variable but a preponderance of countries in this latitudinal zone fell within the ranges <1.5 or 1.6 to 3 μg/d. These low vitamin D supply estimates are unlikely to have a major bearing on population vitamin D status or prevalence of vitamin D deficiency in those countries were exposure to this ample supply of UVB is common, because cutaneous synthesis will ensure adequacy of vitamin D status. As an example, Guatemala, Honduras, and Costa Rica, all clustered within a relatively narrow latitudinal band (~10–15 degrees north) within Meso‐America, have a predicted yearly average UVI of 11.^(^ ^43^ ^)^ For this reason, despite the fact that the three countries have variable average per capita vitamin D supply estimates of 1.7, 1.2 and 3.9 μg/d, respectively, and their mean serum 25(OH)D concentrations in adults and in children are within a narrow range (72–80 nmol/L, and 71–79 nmol/L, respectively), between 0% and 11.5% have serum 25(OH)D <50 nmol/L, and there is an absence of serum 25(OH)D <30 nmol/L.^(^ ^44^ ^)^ On the other hand, if there is an understanding that a population or population subgroup(s), for whatever underlying reason, has a low level of UVB exposure and thus limited cutaneous synthesis of vitamin D, then the food estimates may sound an alarm for risk of high prevalence of vitamin D deficiency. As an example, and staying at a similar latitude band (~12–19 degrees north) and high UVI (10–11), the southern part of India (~12–19 degrees north) has a high reported prevalence of vitamin D deficiency among pregnant women, adults, and very likely children and adolescents at between 19% to 31%.^(^ ^6^ ^)^ A very high prevalence of vitamin D deficiency (using the serum 25(OH)D threshold <25 nmol/L) (43% to 87%) has also been noted for several population subgroups residing at higher latitude in India (eg, Delhi at ~27 degrees north).^(^ ^6^ ^)^ This may relate to low cutaneous synthesis of vitamin D due to skin pigmentation, and cultural and personal practices that lead to avoidance of sun exposure, and environmental factors, such as high degree of air pollution, which lower UVB availability.^(^ ^45^, ^46^ ^)^ The estimated vitamin D supply of ~1 μg/d for India up to 2017 was totally inadequate to prevent the decline in vitamin D status that occurs when UVB‐induced cutaneous synthesis is limited or absent, irrespective of underlying reason. However, the present analyses of 2018 FBS data, and assuming universal adoption of the fortification of milk with the recommended 5 to 7.5 μg/L from the very start of that year rather than August when the regulation was published,^(^ ^16^ ^)^ suggests that per capita vitamin D supply could be increased to ~3 μg/d or more, when one also accounts for fortification of vegetable oil.

Low daily per capita vitamin D supply estimates (0.4–2.5 μg/d) are evident in a number of other countries that reside either wholly or largely within this mid‐latitude zone, and which have been reported to have high burden of vitamin D deficiency; eg, Pakistan, Afghanistan, Yemen, Nigeria, and Tunisia.^(^ ^6^ ^)^ Although a majority of the countries in the 37 degrees north to 37 degrees south latitudinal zone will not have representative serum 25(OH)D data, low vitamin D supply estimates used in conjunction with knowledge of local factors that might impede cutaneous vitamin D synthesis within populations or population subgroup(s) may highlight those countries where assessment of serum 25(OH)D is a priority.

As illustrated by us recently,^(^ ^1^ ^)^ FAO FBS data can also be used to model the potential impact of vitamin D fortification of different food vehicles on the average per capita vitamin D supply in countries where the supply is low and which have a high burden of vitamin D deficiency. Our recent analyses suggest that fortification of wheat, and, to a lesser extent, edible plant‐based oil and milk, with vitamin D at levels which are currently being used within the range of international or nation standards, represents a viable means of improving current low intakes in at‐risk countries. The key role of food fortification with vitamin D in helping address inadequacy of vitamin D intake was also evident in the present work. For example, fortification of milk and dairy produce in the United States, Canada, and Finland provided additional vitamin D in the range 1.5 to 3.2 μg/d over and above that coming from natural food supply, which was itself in the range 4.3 to 7.2 μg/d due to the supply of fish and animal produce. Jordan, Kuwait, United Arab Emirates, and Saudi Arabia, with lower natural food supply of vitamin D (1.5–3.5 μg/d), benefited from the fortification of wheat flour with vitamin D, which was estimated to provide additional vitamin D in the range of 2.9 to 4.4 μg/d. None of these countries had per capita vitamin D supply estimates <5.4 μg/d.

There are a number of limitations to the present work that should be noted. Although the present analyses utilized vitamin D food composition data from the UK McCance and Widdowson's Composition of Foods^(^ ^14^ ^)^ and, to a much lesser extent, the USDA's FDC^(^ ^15^ ^)^ as the most comprehensive and widely used food composition databases, these will fail to include country‐specific and region‐specific differences in vitamin D content of certain foods. For example, we have recently shown that beef derived from cattle slaughtered in different seasons of the year within Ireland have significant differences in vitamin D and 25(OH)D contents.^(^ ^47^ ^)^ Such UVB‐induced differences in meat vitamin D content are likely to exist in countries around the globe that have dramatically different UV availability. The vitamin D content of fish and other animal‐derived produce such as eggs, could also differ in some countries depending on UV availability and animal husbandry practices. It should also be noted again that for some food commodity subcategories, eg, pelagic fish, there can be wide variability in the vitamin D content of individuals species/foods contained within the subcategory. For example, generally the fatty fish within the pelagic subcategory have much higher vitamin D content compared to the white‐type fish.^(^ ^14^ ^)^ Because the FBS only provides an average food supply estimate for each subcategory, and not information on individual foods, the use of an average vitamin D content applied to the average food supply will impact the per capita vitamin D supply estimates, leading to underestimations and overestimations depending on the country. The FBSs only take into account the amount of food available within a country, and as such it has been suggested that nutrient intake may be overestimated owing to wastage from preparation or spoiled foods not being taken into account.^(^ ^48^ ^)^ For vitamin D, however, there were only a few foods that contained vitamin D, and these were of animal origin and possibly less prone to wastage/spoilage. It should also be stressed that the FBS approach cannot account for variations in consumption levels between genders, different population subgroups, or even regional differences, such as urban versus rural.^(^ ^1^ ^)^ In the present work, the vitamin D supply estimates for some countries that fortify edible oils with vitamin D, are underestimates because inclusion of such data was considered too problematic due to many unknowns, as mentioned under methods section. Likewise, the vitamin D supply estimates will be underestimates for some countries in which voluntary fortification of certain foods and/or where practices like commercial UV‐irradiation or sun‐drying of edible mushrooms is common place,^(^ ^49^ ^)^ all of which would contribute to dietary intake. As mentioned under methods section, the level of uptake of voluntary fortification is unclear^(^ ^9^ ^)^ and mushroom supply data is not provided in the FAO FBSs, and thus, could not be taken into account. Finally, the estimates do not capture the contribution that vitamin D–containing supplement use makes to vitamin D supply in some countries at least.

In conclusion, although very cognizant of the limitations of the approach, the per capita daily vitamin D supply estimates nevertheless as surrogate for vitamin D intake data highlight countries and regions where inadequacy of supply may be of concern. It also shows how fortification of food with vitamin D can have an important impact on addressing low vitamin D intake.

## Conflict of Interest

KDC declares no conflict of interest.

### Peer Review

The peer review history for this article is available at https://publons.com/publon/10.1002/jbm4.10547.

## Supporting information


**Appendix** **S1**. Supporting information.
