# Supplementary material for: Global View of Per Capita Daily Vitamin D Supply Estimates as Proxy Measures for Vitamin D Intake Data
Source: JBMR Plus. 2021 Sep 15;5(12):e10547. doi: 10.1002/jbm4.10547 (PMC8674773; doi:10.1002/jbm4.10547)
Supplement: Supplementary file 1 — Appendix S1. Supporting information. [file JBM4-5-e10547-s001.docx]

**Supporting information**

**Supplemental Tables and Figures**

**Supplemental Figure 1**. The per capita supply of pelagic fish in Maldives and of freshwater fish in Myanmar over the period 2004 to 2017.

**Supplemental Figure 2**. The per capita daily supply of all fish combined, all meats combined, eggs and dairy in the Americas, Europe, Africa, Asia and Oceania
